# Association of Dietary Protein Sources and Their Adequacy, Body Composition and Risk of Sarcopenic Obesity in South Korean Populations: A Cross-Sectional Study

**DOI:** 10.3390/metabo14020130

**Published:** 2024-02-19

**Authors:** Jieun Kim, Kyoungsik Jeong, Sueun Lim, Siwoo Lee, Younghwa Baek

**Affiliations:** Division of Korean Medicine Data, Korea Institute of Oriental Medicine, Daejeon 34054, Republic of Korea; jieunkim0218@kiom.re.kr (J.K.); jksik@kiom.re.kr (K.J.); mercury@kiom.re.kr (S.L.); bfree@kiom.re.kr (S.L.)

**Keywords:** body composition, body fat mass, skeletal muscle mass, protein adequacy, sarcopenic obesity

## Abstract

Dietary protein sources and protein adequacy are crucial modulators of muscle quality and body composition. We investigated the association between dietary protein sources (and their adequacy) and body composition and the risk of sarcopenic obesity (SO) in South Korean populations. The participants (*n* = 1967) were classified into SO, obese, sarcopenia, and normal groups. A cross-sectional survey was conducted using the KS-15 questionnaire, short-form food frequency questionnaire, and anthropometric measurements. The percentage of body fat (male: 35.36 ± 0.51%; female: 44.14 ± 0.36%) was significantly high, while appendicular skeletal muscle (ASM; male: 36.39 ± 0.30%, female: 30.32 ± 0.19%) was low in the SO group. Beef and pork consumption was negatively associated with ASM (%) but positively associated with body fat (%) in the normal group and positively associated with ASM (kg/m^2^: beta = 0.002, *p* = 0.02) and BFM (kg: beta = 0.012, *p* = 0.03) in the SO group, respectively. The highest quintile (Q5: 173.6 g/day) showed a decreased risk of SO prevalence (AORs: 0.46, CI: 0.22–0.94) compared with that in the lowest quintile (Q1: 21.6 g/day) among the people with inadequacy protein intake. Daily poultry and egg intake was positively linked with body composition in the participants with SO, while red meat showed a negative effect on imbalanced body composition in participants in the normal and SO groups. Furthermore, a lower intake of poultry and eggs was strongly associated with SO prevalence in people who consumed inadequate amounts of daily dietary protein.

## 1. Introduction

Sarcopenic obesity (SO) is a double-burden condition characterized by a decline in muscular quality and excess body fat mass [1]. SO is caused not only by aging but also by non-communicable diseases, such as cardiovascular diseases (CVDs), metabolic syndrome (MetS), and obesity [2]. Obesity is a risk factor for chronic diseases, including type 2 diabetes mellitus (T2DM), CVDs, and all-cause mortality [3,4,5]. The coexistence of excess adiposity and the loss of muscle mass negatively affects the prognosis of obese individuals ^1^.

As a multifactorial disease, SO is driven by metabolic health status and lifestyle factors, such as diet and physical activity (PA) [6]. Unhealthy lifestyles, including smoking, alcohol consumption, lack of PA, and adherence to an unhealthy diet, showed strong associations with the development of cardiometabolic diseases in a multinational prospective cohort study [7]. Korean medicine (KM) types [8] have a gen11etic predisposition for abdominal obesity [9], insulin resistance [10], metabolic syndrome [11,12], and inflammatory status [13] in various South Korean population-based studies.

Body composition is a reliable predictor of good health status and is evaluated according to fat-free mass and fat mass [14]. Excessive fat accumulation in various non-adipose tissue organs such as skeletal muscle and the liver might be useful in determining the development of a systemic proinflammatory state related to insulin resistance and the progression of impaired skeletal muscle mitochondrial function [15,16]. Obesity alters the adipose tissue in such a way that it secretes a dysregulated production of high levels of proinflammatory cytokines associated with protein degradation, which will eventually lead to the development of metabolic dysregulation and metabolic diseases [17,18]. Therefore, an evaluation of muscle health is suggested in individuals of all age groups.

Dietary protein sources and adequacy are known modifiable risk factors for SO prevalence [19,20]. It has been proposed that plant-based protein food sources, such as fresh fruits, vegetables, dried nuts, flax seeds, whole grains, and soybeans, have the beneficial effects of improving body composition [21], lipid metabolism [22], and cardiovascular health [23]. In contrast, red meat-based diets are reported to have detrimental cardiometabolic effects on adults [24]. It is necessary to prevent these negative effects on muscle health caused by CVDs and comorbidities associated with incongruities between body fat and muscle mass. Therefore, we investigated the association between dietary protein sources (and their adequacy) and the prevalence of SO in South Korean populations, according to their weight status.

## 2. Materials and Methods

### 2.1. Study Setting and Participants

Eligible participants were enrolled in the Korean Medicine Daejeon Citizen Cohort (KDCC) study between 2017 and 2019. KDCC is the first prospective ongoing cohort study that is based on traditional KM to examine established associations between chronic disease and lifestyle risk factors [8]. We performed a cross-sectional survey in 1967 of these study participants using the Korean Sasang constitutional diagnostic questionnaire (KS-15) to identify the KM type and a general health-related questionnaire (supplied by KM).

All data were handled by the web-based Korean Medicine Data Center (KDC) electronic data capture system of the Korean Institute of Oriental Medicine (KIOM). This study was conducted in accordance with the guidelines of the Declaration of Helsinki. Ethical approval was obtained after the participants provided written informed consent. This study was approved by the Ethics Committee of the Korean Institute of Oriental Medicine (IRB No. I-1703/002–002, DJDSKH-17-BM-12).

### 2.2. Definition of Sarcopenia and SO

SO is defined as the coexistence of sarcopenia and obesity which characterized by low skeletal muscle mass and function and excess body fat mass [25]. Sarcopenia is a clinical condition characterized by an age-related loss of muscle mass and muscle strength [26]. We defined sarcopenia as having low appendicular skeletal muscle mass (ASM) in kilograms (kg) [26] which was measured via bioelectrical impedance analysis (BIA, InBody 770, Seoul, South Korea). Muscle strength was assessed by grip strength (kg), using a hand dynamometer (TKK 5401, Takei Scientific Instruments, Niigata, Japan); the maximum reading of two trials was used. Body mass index (BMI) was calculated as weight in kilograms divided by height in meters squared (kg/m^2^).

Sarcopenia

*Muscle Mass (Kg, %)*

The cutoff values of sarcopenia were defined as below the sex-specific mean and standard deviation (SD) for the young reference group (male: 215, female: 375, aged 20–39 years) of the ASM divided by height squared (ASM, kg/m^2^) [26] or ASM as a percentage of body weight (ASM/weight (kg) (%) [26,27]. The cutoff point for sarcopenia was <8.82 kg/m^2^ in men and <6.46 kg/m^2^ in women as measured using ASM (kg/m^2^). For ASM (%), the cutoff was 38.53% in men and 32.16% in women.

*Muscle Strength (Kg)*

Based on the Asian Working Group for Sarcopenia (AWGS) 2019 criteria, a handgrip strength value < 28.0 kg for men and <18.0 kg for women or the lowest quintile of muscle strength among the study participants indicated low muscle strength [26].

Obesity

Obesity was defined as a BMI ≥ 25 kg/m^2^ [28] and the upper two quintiles of the total body fat percentage (%BF) for each sex were used. For females, %BF quintiles were Q1: 25.9, Q2: 29.3–32.7, Q3: 32.8–35.5, Q4: 35.6–38.8, and Q5: 38.9–51.5; for males, these values were Q1: 16.9, Q2: 20.3–23.6, Q3: 23.7–26.3, Q4: 26.4–30.1, and Q5: 30.2–41.3.

Then, we classified participants into SO, obese (OB), sarcopenic (S), and normal groups. The prevalence of sarcopenia (*n* = 81, 4.1%) was similar to the result of the recent nationally representative data (3.8 to 6.5%) in middle-aged adults in Korea [29,30].

### 2.3. Dietary Assessment

The dietary intake of the participants was assessed using a validated short-form food frequency questionnaire [8], which contains 34 food items with serving sizes. Daily food amounts in grams and frequency were calculated from the energy (kcal/day) and macronutrients (carbohydrate, fat, and protein) (g/day) intake using a computer-aided nutritional analysis program (CAN Pro, Version 5.0, the Korean Nutrition Society, 2015). This program is based on the recommended nutritional intake from the Dietary Reference Intake for Koreans (Korean Nutrients Society, 2020). To evaluate the protein adequacy of the individual diet among the participants, dietary protein sources (g/day) were classified into beans and tofu, fish, beef and pork, and poultry and eggs based on the Korean Nutrient Database. Regarding the protein adequacy, participants were categorized into two different groups, adequate: ≥0.8–<1.2 and inadequate: <0.8 or ≥1.2 g/kg/d, according to their protein per kilogram of body weight (g/kg) [31].

### 2.4. Covariates

The participants’ age, sex, lifestyle choices (i.e., smoking status, alcohol consumption, and physical activity level), KM type, and energy intake (kcal/day) were used as covariates in the statistical analysis. The lifestyle components (smoking, drinking and physical activity) were surveyed using a self-reported questionnaire at baseline. Various South Korean population-based studies have reported the KM type to have a genetic predisposition for abdominal obesity, insulin resistance, metabolic syndrome and/or inflammatory status. Individual characteristics of the KM type, such as personality, physiological functions, and symptoms of the participants, were assessed using the KS-15 [32]. The KS-15 is a well-validated, shortened version, and cost-effective screening instrument for assessing the KM type that adapts the BMI and age- and sex-specific weighted values for higher coincidence with clinical relevance (Cronbach a = 0.630) [32]. As previously reported, cardiometabolic outcomes [12] and inflammatory status [13] were assessed according to the two different KM types (Taeeum vs. Non-Taeeum [Soeum or Soyang]) in South Korean adults. Age and energy intake were classified as continuous variables, while sex (male vs. female), smoking (past or not vs. current), drinking (past or not vs. current), physical activity (insufficient vs. sufficient), and KM type (Taeeum vs. Non-Taeeum) were classified as categorical variables.

### 2.5. Statistical Analyses

**Figure 1:**
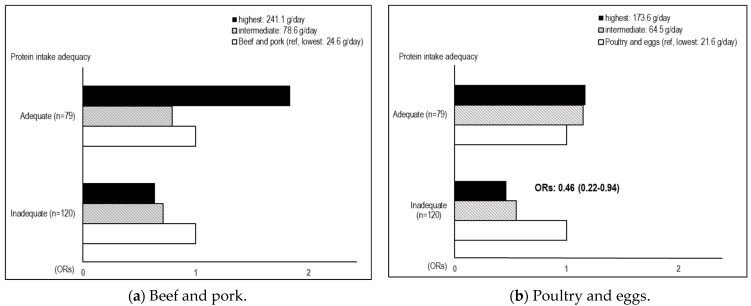
Association between dietary protein sources and sarcopenic obesity based on the adequacy of protein intake. Multivariate logistic regression analysis was performed by adjusting for covariates, including age, sex, energy intake (kcal), smoking, drinking, physical activity, and Korean medicine type. Protein intake adequacy was categorized into three groups according to the mean daily protein intake per body weight as follows: inadequate: <0.8 g/kg/day; adequate: 0.8–1.2 g/kg/day; and excessive: >1.2 g/kg/day. OR: odds ratio; CI: confidence interval; BMI: body mass index.

Frequencies and percentages were used as categorical variables in the descriptive analyses. The chi-square (χ^2^) test was used to compare the general and health-related characteristics (sex, smoking, alcohol consumption, physical activity, and KM type) in the descriptive analysis (Table 1). All data on continuous variables related to body composition, sarcopenia (ASM, kg, kg/m^2^, and %), body fat mass (kg, %) and grip strength (kg) (Table 1), macronutrients (carbohydrates, fat, and protein) intake, and dietary protein sources (beans and tofu, fish, beef and pork, and poultry and eggs) are presented as means ± standard errors (Table 2). Multiple linear regression models were used to examine the association between dietary protein sources, body fat mass (kg, %), ASM (kg/m^2^), and ASM (%) (Table 3). To deal with missing data, multiple imputation was employed to provide unbiased valid estimates of associations based on information from the available data. Multivariate logistic regression was used to evaluate the association between different protein sources (g/day) and the prevalence of sarcopenia or SO, according to the sarcopenia and obesity statuses. Adjusted odds ratios (AORs) and 95% confidence intervals (CIs) were also estimated after adjusting for age, sex, energy intake (kcal), smoking, alcohol consumption, physical activity, and KM type (Table 4). The relationship between dietary protein sources (A. beans and tofu; B. poultry and eggs) and total sarcopenia prevalence was also estimated through multivariate logistic regression based on the protein intake adequacy of the participants (Figure 1). All analyses were performed using SAS version 9.4 (SAS Institute, Inc., Cary, NC, USA). All statistical tests were two-tailed, and *p*-values < 0.05 indicated statistical significance.

## 3. Results

A total of 1967 participants with ages ranging from 30 to 55 years (average age 43.7 ± 0.2 years) were included, and 69.5% (*n* = 1387) of the participants were female individuals. No age group difference was observed between the groups, while the highest ratio of female participants was observed in the normal (*n* = 1018, 74.1%) group. Regarding the KM type, a higher ratio of Taeeum was observed in the SO (*n* = 198, 99.5%) and OB (*n* = 296, 94.6%) groups (*p* < 0.0001). Regarding health-related behaviors, more individuals who smoked and consumed alcohol were observed in the OB group (*p* < 0.0001). Fewer smokers were observed in the normal group (*p* < 0.0001). Body composition statistics showed that body fat mass (kg and %) (male: 32.06 ± 0.50 kg/35.36 ± 0.51%; female: 32.95 ± 0.36 kg/44.41 ± 0.36%) was significantly highest in the SO group compared to the other groups (*p* < 0.001). However, weight-adjusted ASM (%) among individuals of both sexes was the lowest in the SO (male: 36.39 ± 0.30%; female: 30.32 ± 0.19%) group compared to the other groups (*p* < 0.0001) (Table 1).

Table 2 shows the daily energy (kcal/day), macronutrients, and dietary protein sources (g/day) consumed by participants according to their sarcopenia and obesity statuses. No significant differences were observed in the daily intakes of total energy and macronutrients of the participants (both sexes) among the groups. However, protein per kilogram of body weight (g/kg) was the lowest in the SO group (SO: 0.92 ± 0.02) compared to the other groups (OB: 1.00 ± 0.01; S: 1.20 ± 0.02 and normal: 1.20 ± 0.01) (*p* < 0.0001) (Table 2).

The association between dietary protein sources and body composition of the participants according to their sarcopenia and obesity statuses is presented in Table 3. There was no association between beans and tofu, and poultry and eggs (g/day), and body composition (ASM [kg/m^2^ and %], BFM, and PBF) among the participants. The consumption of beef and pork was positively associated with ASM (kg/m2: beta = 0.002, *p* = 0.02) and BFM (kg: beta = 0.012, *p* = 0.03) in the SO group, while it was positively associated with PBF (%: beta = 0.003, *p* = 0.02) and negatively associated with ASM (%: beta = −0.002, *p* = 0.01) in the normal group, respectively (Table 3).

The number of participants with SO (*n* = 199) and sarcopenia (*n* = 81) is presented in Table 4. Multivariate logistic regression was performed by adjusting for covariates, including age, sex, energy intake (kcal), smoking, alcohol consumption, PA, and KM type (Table 4). According to the quintiles of poultry and eggs intake (g/day), a negative association of the SO prevalence (AORs: 0.52, CI: 0.30–0.90) was shown in the highest quintile (Q5: 173.6 g/day) compared with that in the lowest quintile (Q1: 21.6 g/day) (Table 4).

Regarding the protein sources and adequacy and the SO prevalence, the highest (Q5: 173.6 g/day) quintiles of poultry and eggs intake were associated with a great decreased risk of SO (AORs: 0.46, CI: 0.22–0.94) in the inadequate protein intake groups, compared with the lowest quintile (Q1: 21.6 g/day) (Figure 1).

## 4. Discussion

We examined the association of dietary protein sources and protein adequacy, body composition, and prevalence of SO in South Korean populations. With regard to red meat (beef and pork) consumption, it was negatively associated with ASM (%) but positively associated with PBF (%) in the normal group. Similarly, a positive association between red meat consumption and ASM (kg/m^2^) and BFM (kg) was observed in the participants with SO. Oppositely, it was found that a higher consumption of poultry and eggs among people who consumed inadequate amounts of daily dietary protein was related to lower SO prevalence.

Obesity Risk Factors in Sarcopenic Obesity Prevalence

Our results are consistent with those of other studies on sarcopenia in the South Korean population [33,34] using the ASM (%) to identify the prevalence of SO. Obesity is a low chronic inflammation factor that may lead to the development of SO via the infiltration of lipid deposition in the muscle tissue from extensive fat accumulation [35]. In line with this, the combination of sarcopenia and obesity more significantly accelerates deteriorating muscle health when compared with the entities associated with chronic disease states in South Korean adults [36]. Similarly, our results showed higher risks of body composition changes and the prevalence of SO in the participants.

A previous study reported decreased lean body mass and increased fat mass in the overweight/obese group, compared with healthy lean controls [37]. There were positive associations with intermuscular fat mass and high-sensitivity C-reactive protein (hs-CRP) and cortisol concentrations, as well as a negative association with SMM [37]. Similarly, the SO and OB groups included more Taeeum-type individuals with predisposing metabolic risk factors [12,13], compared with the BMI < 25 kg/m^2^ group. Our cross-sectional results from a previous cohort study showed associations with a higher prevalence of pre-MetS and higher hs-CRP levels in Taeeum-type South Korean populations [12,13]. This reflects obesity-related physiological characteristics associated with the Taeeum-type, which can be positively associated with SO prevalence in people who are obese. Contributing lifestyle factors include the consumption of uncontrolled or Western-type diets and physical inactivity or sedentary lifestyles, which lead to imbalanced inflammatory mediators stimulating muscle tissues that aggravate low-grade inflammation [38].

Effects of Dietary Protein Sources and Adequacy on Muscle Physiology and Body Composition

Dietary protein ingestion is a key modulator of muscle mass and function [39]. Furthermore, it has favorable effects on weight management and maintaining a cardiometabolic profile [40]. A result from a large prospective cohort study demonstrated that high animal protein, especially that from processed red meat, was positively associated with cardiovascular mortality among individuals with at least one lifestyle risk factor in 131,342 participants [41]. In our previous study, we found an approximately 2-fold higher meat intake increase in people with SO (PR: 1.93, 95% CI: 1.07–3.50), compared with that of the lowest tertile in older South Koreans with cardiometabolic diseases [40]. Correspondingly, we found that the consumption of beef and pork was negatively related to ASM (%) but positively related to PBF (%) in the normal group. Meanwhile, positive associations were observed between the intake of red meats and ASM (kg/m^2^) and body fat mass (kg) in the participants with SO in the present study.

It was recommended that a substitution of red and processed meats with poultry or fish should take place in a systematic review study [42]. In overweight and obese women who were in the upper category of the DASH dietary pattern, the risk of SO was reduced by 80% (OR = 0.20, 95% CI = 0.05 to 0.77, *p* = 0.01) in another study [43]. Correspondingly, our results showed that participants who consumed more than 173.6 g/day of poultry and eggs reported a reduced the risk of developing SO by 48% compared with those in the lowest intake group (Q1: 21.6 g/day). Similarly, it was also shown that people with the inadequate protein intake (<0.8 or >1.2 g/kg/day) among the individuals who had the highest intake of poultry and eggs had a reduced risk of developing SO by 54% compared with that of the lowest intake group. However, it was also shown that the consumption of poultry and eggs and fish was negatively related to ASM (%) but not beans and tofu in the normal group who had an excess protein diet. Contrariwise, it was reported that a hypocaloric diet (500 kcal deficit) with either normal (0.8 g/kg/d) or higher protein (1.2 g/kg/d; predominantly from lean red meat) improves cardiometabolic outcomes and insulin resistance in obese adults in another study [44]. Based on these results, it could be assumed that the dietary protein sources and adequacy might be problematic dietary risk factors according to individual body composition and weight statuses.

Dietary protein digestibility and digested meat products vary with the animal source which affects protein digestion in the digestive tract [45]. The peptide profiling, which is caused by pepsin and trypsin cleavages, of chicken and fish is quite different, while pork and beef have great similarity [45]. Furthermore, meat and meat products digestion and metabolization contribute to individual body oxidation statuses [46]. Animal meat is one of the major excellent sources of human food. Nevertheless, non-meat foods (plant-based proteins such as beans and tofu, fish and seafoods) contain much fewer saturated fats, are free of dietary cholesterol, and even provide adequate amounts of minerals (phosphorus, iron, and zinc) when compared with meat products [47]. Therefore, an adequate amount of high-quality protein, supplementation with more plant-based foods, such as legumes, beans and nuts, a low-saturated fat diet, and avoiding cooking with energy-rich ingredients could reduce the risk of underlying health conditions in mid- and later-life.

Strength and Limitations

To the best of our knowledge, this is the first study to evaluate the associations between SO prevalence and dietary protein sources by considering the predominant personality type with complex and different lifestyles, including diet, using the KM type. We also used valid and reliable nationally representative, multilayer sampling data from the KDCC study. Finally, a wide range of covariates were considered in the adjustment of the multivariate analyses, including age, sex, health-related behaviors (smoking status, alcohol consumption, and physical activity level), KM type, and energy intake (kcal/day). These methods yielded descriptive results which greatly enhanced our understanding of SO prevalence and risk factors.

This study has some limitations. First, this cross-sectional study cannot provide causal inferences due to the limited evidence. Second, we did not consider the residual confounding factors, such as genetic or medical history, as covariates. We also employed to ASM (%) or ASM (kg/m^2^) cut-off values to define sarcopenia, following previous sarcopenic studies in Korea [31,43,44,45]. To date, no consensus definition has been universally adopted to define sarcopenic obesity in the middle-aged population. Therefore, further studies which use a standardized definition of sarcopenic obesity are necessary to include muscle function and quality and physical performance which use a standardized definition of sarcopenia. Lastly, reverse causality might exist because the individuals with SO tended to consume fewer protein sources than their counterparts. However, there was no variation in dietary intake between groups according to the status of sarcopenia and obesity in the participants; however, protein (g/kg) consumption of the participants varied between groups. The reason could be that people with higher BMI values did not consume adequate and/or high-quality protein relative to their body weight. It is also predicted that they consumed higher percentages of fat- or carbohydrate-rich foods in their diet. Therefore, careful interpretation is required to ensure the generalizability of our findings.

## 5. Conclusions

In conclusion, dietary protein sources and adequacy are strongly related to body composition in South Korean populations. Therefore, optimal dietary choices and adequate intake of high-quality protein are essential to maintain muscle health in people who follow an obesogenic lifestyle with an unhealthy diet.

## Figures and Tables

**Table 1:** General characteristics and body composition of the participants according to the status of sarcopenia and obesity ^†^.

General Characteristics	SO (*n* = 199)	OB (*n* = 313)	S (*n* = 81)	Normal (*n* = 1374)	*p*-Value
Age (years) (mean ± SE)					0.279
30–39 years (*n*, %)	71 (35.7)	87 (27.8)	24 (29.6)	304 (29.7)	
40–55 years	128 (64.3)	226 (72.2)	57 (70.4)	509 (70.3)	
Sex (%)					<0.0001
Male	68 (34.2)	152 (48.6)	27 (33.3)	356 (25.9)	
Female	131 (65.8)	161 (51.4)	54 (66.7)	1018 (74.1)	
KM type ^1^ (%)					<0.0001
Taeeum	198 (99.5)	296 (94.6)	49 (60.5)	457 (33.3)	
Soeum	0 (0.0)	0 (0.0)	14 (17.3)	375 (27.3)	
Soyang	1 (0.5)	17	(5.4)	18 (22.2)	
Smoking (%)					<0.0001
Past or not	150 (75.4)	218 (69.6)	63 (77.8)	1133 (82.5)	
Current	49 (24.6)	95 (30.4)	18 (22.2)	241 (17.5)	
Drinking (%)					0.07
Past or not	69 (34.7)	111 (35.5)	36 (44.4)	570 (41.5)	
Current	130 (65.3)	202 (64.5)	45 (55.6)	804 (58.5)	
Physical activity ^2^ (%)					0.598
Insufficient	131 (65.8)	217 (69.3)	59 (72.8)	922 (67.1)	
Sufficient	68 (34.2)	96 (30.7)	22 (27.2)	452 (32.9)	
Body composition					
ASM, kg/m^2^					
Male	11.14 ± 0.09 ^b^	11.51 ± 0.06 ^a^	9.14 ± 0.15 ^c^	10.51 ± 0.04 ^c^	<0.0001
Female	9.08 ± 0.06 ^b^	9.31 ± 0.05 ^a^	7.78 ± 0.09 ^d^	8.14 ± 0.02 ^c^	<0.0001
ASM, %					
Male	36.39 ± 0.30 ^c^	41.18 ± 0.20 ^b^	40.85 ± 0.47 ^b^	43.82 ± 0.13 ^a^	<0.0001
Female	30.32 ± 0.19 ^d^	33.99 ± 0.18 ^b^	31.63 ± 0.30 ^c^	36.65 ± 0.07 ^a^	<0.0001
Body fat mass, kg					
Male	32.06 ± 0.50 ^a^	23.03 ± 0.34 ^b^	17.42 ± 0.80 ^c^	15.78 ± 0.22 ^c^	<0.0001
Female	32.95 ± 0.36 ^a^	26.60 ± 0.32 ^c^	24.77 ± 0.56 ^b^	17.98 ± 0.13 ^d^	<0.0001
Body fat mass, kg					
Male	35.36 ± 0.51 ^a^	27.33 ± 0.34 ^b^	26.33 ± 0.81 ^b^	22.16 ± 0.22 ^c^	<0.0001
Female	44.14 ± 0.36 ^a^	37.78 ± 0.32 ^c^	40.66 ± 0.56 ^b^	31.84 ± 0.13 ^d^	<0.0001
BMI, kg/m ^2§^	30.50 ± 0.16 ^a^	27.71 ± 0.13 ^b^	24.27 ± 0.25 ^c^	23.04 ± 0.07 ^d^	<0.0001

^†^ The status of sarcopenia and obesity were defined by body composition (reduced muscle mass (kg and %) and increased %BF) after screening elevated body mass index (BMI) of the participants. ^a–d^ The different letters indicate statistically significant differences (*p* < 0.05), analyzed using ANCOVA followed by Bonferroni’s multiple comparison test. ^§^ The least square is presented as means ± SEs adjusted for age and sex (age only adjusted for body composition). ^1^ KM (Korean medicine) type was categorized into three groups: Taeeumin, Soeumin, and Soyangin. ^2^ Physical activity (total MET minutes per week): insufficient (<600 MET min/week) and sufficient (≥600 MET min/week). BMI: body mass index, ASM: appendicular skeletal muscle. SO: Sarcopenic obesity; S: sarcopenia; OB: obesity.

**Table 2:** Group differences in dietary intake according to the status of sarcopenia and obesity of the participants.

Dietary Intake and Protein Sources	SO (*n* = 199)		OB (*n* = 313)		S (*n* = 81)		Normal (*n* = 1374)		*p*-Value
Energy intake (kcal/day) ^1^									
Male	2201.81	±79.89	2253.52	±53.44	2182.85	±126.80	2274.73	±34.91	0.782
Female	2129.64	±60.66	2079.72	±54.82	2017.14	±94.65	2063.95	±27.77	0.708
Macronutrients (g/day)									
Carbohydrates (g)	317.99	±2.99	316.69	±2.41	321.21	±4.69	319.71	±1.14	0.658
Fat (g)	52.76	±0.99	54.00	±0.80	53.56	±1.55	52.80	±0.38	0.569
Protein (g)	71.46	±0.73	72.57	±0.59	72.30	±1.14	71.52	±0.28	0.398
Protein (g/kg)	0.92	±0.02 ^c^	1.00	±0.01 ^b^	1.20	±0.02 ^a^	1.20	±0.01 ^a^	<0.0001
C: F: P (%)	59.8: 22.2: 13.4		59.5: 22.8: 13.6		60.2: 22.7: 13.5		60.2: 22.3: 13.4		N/S
Dietary protein sources (g/day) ^2^									
Beans and tofu	31.08	±2.52	34.93	±2.03	29.31	±3.95	34.41	±0.97	0.370
Poultry and eggs	85.34	±6.37	93.77	±5.15	81.35	±9.99	85.90	±2.43	0.293
Beef and pork	102.29	±7.19	108.37	±5.80	112.91	±11.28	109.58	±2.75	0.793
Fish	5.70	±0.59	6.04	±0.48	5.73	±0.93	6.34	±0.23	0.671

^1^ Adjusted for age alone. ^2^ The least square is presented as means ± SEs adjusted for age, sex, and energy intake (kcal). BMI: body mass index. ^a–d^ The different letters indicate statistically significant differences (*p* < 0.05), analyzed using ANCOVA followed by Bonferroni’s multiple comparison test.

**Table 3:** Associations between dietary protein sources and body composition of the participants according to their sarcopenia and obesity statuses.

	SO (*n* = 199)		OB (*n* = 313)		S (*n* = 81)		Normal (*n* = 1374)	
Protein Sources (g/Day)	*Beta*	*p*-Value	*Beta*	*p*-Value	*Beta*	*p*-Value	*Beta*	*p*-Value
Beans and tofu								
ASM, kg/m^2^	0.001	0.68	0.000	0.80	0.000	0.80	−0.002	0.60
ASM, %	0.002	0.62	−0.004	0.07	−0.008	0.59	−0.003	0.13
BFM, kg	0.004	0.78	0.006	0.22	0.006	0.81	0.005	0.06
PBF, %	−0.002	0.78	0.008	0.05	0.014	0.62	0.005	0.14
Poultry and eggs								
ASM, kg/m^2^	0.000	0.23	0.000	0.97	0.000	0.74	0.000	0.92
ASM, %	0.001	0.37	0.001	0.57	0.004	0.59	−0.002	0.03
BFM, kg	0.001	0.76	0.000	0.90	−0.002	0.86	0.002	0.13
PBF, %	−0.001	0.56	−0.001	0.67	−0.004	0.72	0.003	0.05
Beef and pork								
ASM, kg/m^2^	0.002	0.02	0.000	0.81	0.000	0.91	−0.001	0.35
ASM, %	0.000	0.80	0.000	0.59	0.001	0.73	−0.002	0.01
BFM, kg	0.012	0.03	0.000	0.94	−0.002	0.68	0.002	0.07
PBF, %	0.001	0.80	0.000	0.74	−0.002	0.79	0.003	0.02
Fish								
ASM, kg/m^2^	−0.014	0.14	−0.003	0.61	0.008	0.58	−0.002	0.22
ASM, %	0.009	0.54	0.009	0.52	−0.146	0.04	−0.016	0.03
BFM, kg	−0.060	0.32	−0.030	0.31	0.203	0.06	0.001	0.21
PBF, %	−0.019	0.49	−0.023	0.37	0.263	0.04	0.025	0.06

Adjusted for age, sex, BMI, energy intake (kcal), smoking, alcohol consumption, physical activity, and Korean medicine type. Statistical significance was accepted at *p* < 0.05. BMI: body mass index, ASM: appendicular skeletal muscle, BFM: body fat mass, PBF: percent body fat.

**Table 4:** Multivariate logistic regression analysis of the association between different protein sources (g/day) and prevalence of SO and sarcopenia.

	SO (*n* = 199)	S (*n* = 81)
Dietary protein sources		
Beans and tofu		
(ref, lowest: 7.1 g/day)	ORs (CI)	
intermediate: 20.5 g/day	0.74 (0.45–1.20)	0.79 (0.40–1.58)
highest: 74.1 g/day	0.56 (0.29–1.09)	0.87 (0.41–1.84)
Poultry and eggs		
(ref, lowest: 21.6 g/day)	ORs (CI)	
intermediate: 64.5 g/day	0.62 (0.37–1.02)	1.05 (0.49–2.23)
highest: 173.6 g/day	0.52 (0.30–0.90)	0.95 (0.40–2.24)
Beef and pork		
(ref, lowest: 24.6 g/day)	ORs (CI)	
intermediate: 78.6 g/day	0.68 (0.41–1.13)	1.09 (0.57–2.08)
highest: 241.1 g/day	0.83 (0.46–1.49)	0.92 (0.40–2.12)
Fish		
(ref, lowest: 0.7 g/day)	ORs (CI)	
intermediate: 3.5 g/day	0.84 (0.49–1.43)	0.81 (0.41–1.60)
highest: 16.2 g/day	0.84 (0.48–1.46)	0.67 (0.32–1.42)

Age, sex, energy intake (kcal), smoking, drinking, physical activity, and Korean medicine type were adjusted. OR: odds ratio; CI: confidence interval.

## Data Availability

The datasets are not available due to confidentiality and ethical concerns. Further inquiries can be directed to the corresponding author.
